# Course of Hyperkalemia in Patients on Hemodialysis

**DOI:** 10.1155/2022/6304571

**Published:** 2022-04-28

**Authors:** Bruce Spinowitz, Steven Fishbane, Masafumi Fukagawa, Martin Ford, Nicolas Guzman, Anjay Rastogi

**Affiliations:** ^1^Department of Medicine, NewYork-Presbyterian Queens, Queens, NY, USA; ^2^Department of Medicine, Donald and Barbara Zucker School of Medicine at Hofstra/Northwell, Great Neck, NY, USA; ^3^Division of Nephrology, Endocrinology and Metabolism, Department of Internal Medicine, Tokai University School of Medicine, Isehara, Japan; ^4^Department of Renal Medicine, King's College Hospital NHS Trust, London, UK; ^5^Faculty of Life Sciences and Medicine, King's College London, London, UK; ^6^Global Medicines Development, AstraZeneca BioPharmaceuticals Research and Development, Gaithersburg, MD, USA; ^7^UCLA CORE Kidney Program, University of California Los Angeles, Los Angeles, CA, USA

## Abstract

**Background:**

Evidence of longitudinal serum potassium (sK^+^) concentrations in hyperkalemic hemodialysis patients is sparse.

**Objective:**

These post hoc analyses of the placebo arm of the phase 3b DIALIZE study (NCT03303521) explored the course of hyperkalemia in hemodialysis patients receiving placebo.

**Methods:**

In DIALIZE, 196 patients receiving hemodialysis three times weekly were randomized to placebo or sodium zirconium cyclosilicate 5 g starting dose once daily on nondialysis days for 8 weeks. In these post hoc analyses of placebo patients overall (*n* = 86) and by predialysis sK^+^ subgroups at randomization <5.5 mmol/L, 5.5 to <6.0 mmol/L, 6.0 to <6.5 mmol/L, and ≥6.5 mmol/L, we assessed mean predialysis sK^+^ concentration by visit and the proportions of patients with mean predialysis sK^+^ ranges of 4.0–5.0 and 4.0–5.5 mmol/L by visit.

**Results:**

In placebo patients, the mean predialysis sK^+^ concentration at randomization was 5.9 mmol/L, and 5.8 mmol/L at the end of the study (day 57). For placebo patients overall and across all predialysis sK^+^ subgroups, the mean predialysis sK^+^ concentration remained ≥5.0 mmol/L for all visits over 8 weeks. Overall, 7–21% and 27–62% of placebo patients had predialysis sK^+^ ranges of 4.0–5.0 and 4.0–5.5 mmol/L, respectively, at any visit. The proportions of placebo patients with either predialysis sK^+^ range were greatest for those who were least hyperkalemic (<5.5 mmol/L) and generally decreased with increasing predialysis sK^+^ concentration.

**Conclusions:**

Patients receiving placebo and hemodialysis maintained high predialysis sK^+^ concentrations over 8 weeks following a hyperkalemic event. Most placebo patients remained hyperkalemic and may be at continued risk of adverse events.

## 1. Introduction

Patients with end-stage renal disease (ESRD) have reduced renal potassium excretion and require dialysis to remove accumulated potassium. Hyperkalemia in hemodialysis (HD) is common [1, 2], and predialysis serum potassium (sK^+^) concentration >5.5 mmol/L is associated with increased mortality and hospitalization [3–6].

The extent of hyperkalemia and variation in sK^+^ concentration in HD patients depends on several variables, including the frequency, duration, and prescription of HD [1]. Patients accumulate potassium between dialysis treatments and sK^+^ concentration rises toward predialysis concentrations [7]. In one analysis of 14 patients receiving standardized 4-hour HD three times weekly (TIW), the mean potassium concentration declined from 5.65 mmol/L predialysis to 3.62 mmol/L postdialysis, but it returned to 5.01 mmol/L at 6 hours postdialysis and 5.6 mmol/L at 38 hours postdialysis (i.e., the interval to the next HD) [8].

Evidence of longitudinal trajectory of sK^+^ concentration and risk of recurrent hyperkalemia following a hyperkalemic event is sparse [9–14], particularly among HD patients [1]. The frequency of potassium monitoring in clinical practice is typically performed based on the clinician's judgement and varies across regions [10]. Nephrologists typically monitor predialysis sK^+^ concentration monthly in HD patients [7, 15]. Evaluation of the longitudinal trajectory of sK^+^ concentration and risk of recurrent hyperkalemia following a hyperkalemic event is needed to inform physicians of the likely course and burden of hyperkalemia in HD patients, and to provide more information regarding the need for impact management strategies.

The phase 3b DIALIZE study (NCT03303521) [16] evaluated sodium zirconium cyclosilicate (SZC) compared with placebo in the management of hyperkalemia in HD patients. The placebo arm of this clinical trial provides the opportunity to evaluate the trajectory of sK^+^ concentration in hyperkalemic HD patients who received placebo in addition to maintenance HD. Here, we report the results of post hoc analyses of the placebo arm from the phase 3b DIALIZE study to examine the course of hyperkalemia in patients who received placebo.

## 2. Materials and Methods

### 2.1. Study Design and Patients

DIALIZE was an international, randomized, double-blind, placebo-controlled phase 3b study; the full details have been reported previously [16]. The study consisted of a 1-week screening period, an 8-week treatment period comprising 4 weeks for dose titration and 4 weeks for evaluation on stable dose, and a 2-week follow-up period. Patients were randomized 1:1 in a blinded manner to orally receive a starting dose of 5 g of SZC or placebo once daily on nondialysis days (4 days/week). All patients received HD TIW and routine dietary counselling as per local guidelines, including dietary potassium restriction. Management of dialysis prescription was implemented according to local clinical practices. Rescue therapy in accordance with local practice patterns to reduce sK^+^ in the setting of severe hyperkalemia (>6.0 mmol/L) included potassium binders, acute potassium-lowering agents, additional dialysis sessions, and a reduction in dialysate potassium (dK^+^) concentration.

Eligible patients were adults aged ≥18 years with ESRD who were managed for ≥3 months before randomization by HD TIW and had a prescribed dK^+^ concentration of ≤3.0 mmol/L at screening. During screening, patients were required to have hyperkalemia despite maintenance HD, defined as predialysis sK^+^ >5.4 mmol/L after the long interdialytic interval (LIDI) on day −7 and predialysis sK^+^ >5.0 mmol/L after at least one short interdialytic interval (SIDI) on days −5 and −3. Patients were required to have sustained blood flow (Qb) ≥200 mL/min and single-pool Kt/V (spKt/V) ≥1.2 (or urea reduction ratio [URR] ≥63%) on stable HD during screening, with prescription expected to remain unchanged during the study.

### 2.2. Data Collection

Central laboratory sK^+^ samples were obtained throughout the study (both predialysis and postdialysis at visits after the LIDI and only predialysis for visits after the SIDI). Parameters of dialysis adequacy (target spKt/V and/or URR) and prescription (dialysate flow [Qd], dK^+^ concentration, prescribed ultrafiltration, and Qb) were recorded longitudinally. Study sites consistently used either spKt/V or URR in determining dialysis adequacy.

### 2.3. Post Hoc Analyses

In these post hoc analyses, we assessed biochemical outcomes in placebo patients. Analyses included predialysis and postdialysis sK^+^ concentrations and shift in sK^+^ concentration from predialysis to postdialysis at each study visit during the dose-titration and 4-week evaluation periods. Predialysis sK^+^ concentrations were available at all LIDI visits (days 1, 8, 15, 22, 29, 36, 43, 50, and 57) and SIDI visits (days 3, 5, 10, 12, 17, 24, 31, 38, 45, and 52); postdialysis sK^+^ concentrations and shift in sK^+^ concentration from predialysis to postdialysis were available at all LIDI visits and some SIDI visits during the dose-titration period (days 3, 5, and 12). Furthermore, we assessed the proportions of patients at each LIDI and SIDI visit who had a prespecified predialysis sK^+^ range of 4.0–5.0 mmol/L and an extended range of 4.0–5.5 mmol/L, and the number of LIDI visits (≥1, ≥2, ≥3, and 4) during the 4-week evaluation period (days 36, 43, 50, and 57), in which patients had sK^+^ concentrations that fell within these ranges. The extended predialysis sK^+^ range of 4.0–5.5 mmol/L reflected one that was deemed to be acceptable in clinical practice, as predialysis sK^+^ concentrations >5.5 mmol/L are associated with increased hospitalization and mortality [3, 5, 6]. To assess the risk of recurrent hyperkalemia, we assessed the incidence of predialysis sK^+^ concentrations >5.5 or >6.0 mmol/L at LIDI visits during the 4-week evaluation period. Parameters of dialysis prescription and adequacy are reported. Concentrations of dK^+^ were available at days −7, −5, −3, 1, 3, 5, 8, 12, 15, 22, 29, 36, 43, 50, and 57; dialysis adequacy parameters were available at days −7, 29, and 57; and dialysis prescription parameters were available at days −7, 1, 29, and 57. Shift from baseline (visit 1, day −7) to the end of treatment (visit 15, day 57) in the proportions of patients with dK^+^ concentration categories of 1, 2, and 3 mmol/L is reported.

### 2.4. Statistical Analysis

Analyses were stratified by predialysis sK^+^ concentration at randomization (visit 4, day 1): <5.5 mmol/L, 5.5 to <6.0 mmol/L, 6.0 to <6.5 mmol/L, and ≥6.5 mmol/L. Baseline for subgroup stratification was defined as the last visit before study drug initiation (i.e., randomization visit) to account for variation from screening in predialysis sK^+^ concentration and to assess patients with a predialysis sK^+^ concentration of <5.5 mmol/L. No imputation of missing data was conducted. Concentration of sK^+^ and shift in sK^+^ concentration from predialysis to postdialysis at study visits are reported as mean (standard deviation [SD]) and range. The proportions of patients with the two ranges of predialysis sK^+^ at study visits are reported descriptively; patients receiving rescue therapy were included in the analyses. For each predialysis sK^+^ subgroup, the incidence of predialysis sK^+^ >5.5 or >6.0 mmol/L at subsequent LIDI visits during the 4-week evaluation period was calculated as follows:

  [Number of hyperkalemic events/total number of possible hyperkalemic events based on patients with evaluable data]× 100

Parameters of dialysis prescription and adequacy are reported as mean (SD).

## 3. Results

### 3.1. Patients

Overall, 99 patients were randomized to receive placebo. Of these, 86 had predialysis sK^+^ measurements at randomization (visit 4, day 1); data were missing for 13 patients. Among these 86 placebo patients, the baseline (visit 1, day −7) mean (SD) age was 60.3 (13.2) years, with 45.3% of patients aged 65–84 years, mean (SD) body mass index was 26.7 (5.5) kg/m^2^, 41.9% of patients were female, and the mean (SD) predialysis sK^+^ concentration was 6.0 (0.4) mmol/L (Table 1). At baseline, most patients had hypertension (80.2%) and 25.6% had type 2 diabetes; 14.0% and 26.7% of patients were receiving concomitant angiotensin-converting enzyme inhibitors and angiotensin II antagonists, respectively, during study treatment (Table 1). Mean (SD) dialysis parameters at baseline were dK^+^ concentration2.2 (0.5) mmol/L, spKt/V 1.7 (0.4), and Qd 548.0 (131.1) mL/min, and most patients (73.3%) had a dK^+^ concentration of 2 mmol/L (Table 1). Overall, 14, 37, 21, and 14 patients had predialysis sK^+^ concentrations at randomization of <5.5 mmol/L, 5.5 to <6.0 mmol/L, 6.0 to <6.5 mmol/L, and ≥6.5 mmol/L, respectively, measured after the LIDI.

### 3.2. Predialysis sK^+^

**Figure 1:**
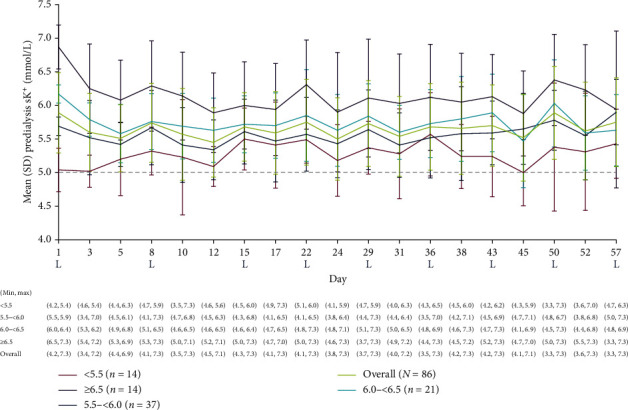
Predialysis sK^+^ concentration at each study visit in placebo patients overall and by predialysis sK^+^ concentration at randomization. Predialysis sK^+^ measurement at randomization (visit 4, day 1) was used for subgroup stratification. No imputation of missing data was conducted. The dashed line represents the upper limit of normokalemia (i.e., sK^+^ 5.0 mmol/L). L represents the LIDI visits. LIDI, long interdialytic interval; SD, standard deviation; sK^+^, serum potassium.

In placebo patients overall (*n* = 86) and across all randomization predialysis sK^+^ subgroups, the mean predialysis sK^+^ concentration remained ≥5.0 mmol/L for all study visits (Figure 1). In placebo patients overall, the mean (SD) predialysis sK^+^ concentration at the end of the study (visit 15 [LIDI], day 57) was 5.8 (0.7) mmol/L. Among the randomization predialysis sK^+^ subgroups of <5.5 mmol/L, 5.5 to <6.0 mmol/L, 6.0 to <6.5 mmol/L, and ≥6.5 mmol/L, a mean (SD) predialysis sK^+^ concentration at end of study was 5.4 (0.5) mmol/L, 5.9 (0.5) mmol/L, 5.6 (0.5) mmol/L, and 5.9 (1.2) mmol/L, respectively.

**Figure 2:**
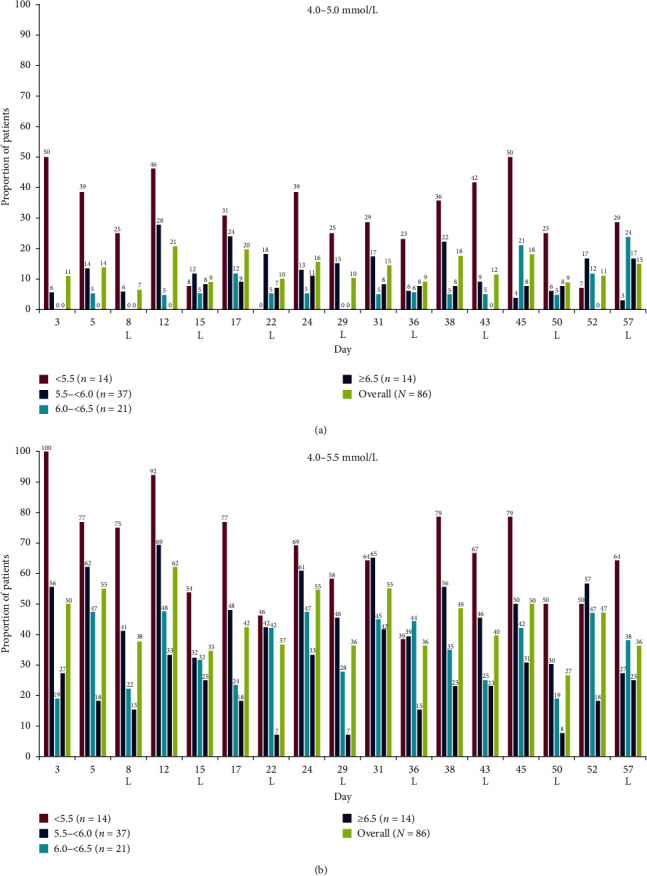
Proportions of placebo patients with predialysis sK^+^ ranges of (a) 4.0–5.0 mmol/L and (b) 4.0–5.5 mmol/L at each study visit, by predialysis sK^+^ concentration at randomization. Predialysis sK^+^ measurement at randomization (visit 4, day 1) was used for subgroup stratification. Patients receiving rescue therapy were included in the analysis. No imputation of missing data was conducted. L represents the LIDI visits. LIDI, long interdialytic interval; sK^+^, serum potassium.

The proportions of placebo patients who exhibited a predialysis sK^+^ range of 4.0–5.0 mmol/L and an extended range of 4.0–5.5 mmol/L at study visits were also assessed. The proportions of placebo patients with a predialysis sK^+^ range of 4.0–5.0 mmol/L and extended range of 4.0–5.5 mmol/L were greatest for those who were least hyperkalemic (i.e., <5.5 mmol/L) at randomization, and generally decreased with increasing randomization predialysis sK^+^ concentration (Figures 2(a) and 2(b)).

**Figure 3:**
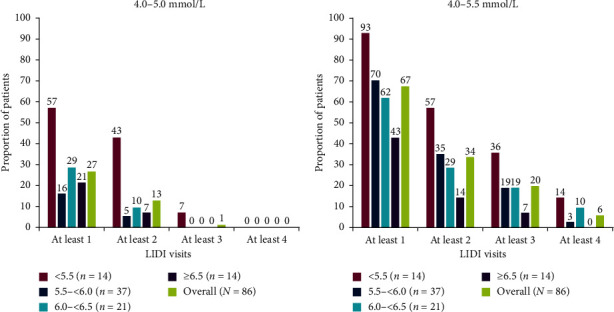
Proportion of placebo patients with predialysis sK^+^ ranges of 4.0–5.0 and 4.0–5.5 mmol/L for ≥1, ≥2, ≥3, and 4 LIDI visits during the 4-week evaluation period, by predialysis sK^+^ concentration at randomization. Predialysis sK^+^ measurement at randomization (visit 4, day 1) was used for subgroup stratification. Predialysis sK^+^ values obtained at the LIDI visits in the 4-week evaluation period. No imputation of missing data was conducted. Patients receiving rescue therapy were included in the analysis. LIDI, long interdialytic interval; sK^+^, serum potassium.

Overall, 27% of all placebo patients (*n* = 86) had a predialysis sK^+^ concentration of 4.0–5.0 mmol/L at ≥1 of the four LIDI visits during the 4-week evaluation period (Figure 3). Meanwhile, 57%, 16%, 29%, and 21% of patients in the randomization predialysis sK^+^ subgroups of <5.5 mmol/L, 5.5 to <6.0 mmol/L, 6.0 to <6.5 mmol/L, and ≥6.5 mmol/L, respectively, had a predialysis sK^+^ concentration of 4.0–5.0 mmol/L at ≥1 of the four LIDI visits during the 4-week evaluation period (Figure 3). No placebo patient overall (*n* = 86) had a predialysis sK^+^ concentration of 4.0–5.0 mmol/L at all four LIDI visits (Figure 3).

Overall, 67% of all placebo patients (*n* = 86) had a predialysis sK^+^ concentration within the extended range of 4.0–5.5 mmol/L at ≥1 of the four LIDI visits during the 4-week evaluation period; corresponding proportions were 93%, 70%, 62%, and 43% in the randomization predialysis sK^+^ subgroups of <5.5 mmol/L, 5.5 to <6.0 mmol/L, 6.0 to <6.5 mmol/L, and ≥6.5 mmol/L, respectively (Figure 3). Meanwhile, 6% of all placebo patients (*n* = 86) had a predialysis sK^+^ concentration of 4.0–5.5 mmol/L at all four LIDI visits; the proportions in the randomization predialysis sK^+^ subgroups of <5.5 mmol/L, 5.5 to <6.0 mmol/L, 6.0 to <6.5 mmol/L, and ≥6.5 mmol/L were 14%, 3%, 10%, and 0%, respectively (Figure 3).

### 3.3. Postdialysis sK^+^

**Figure 4:**
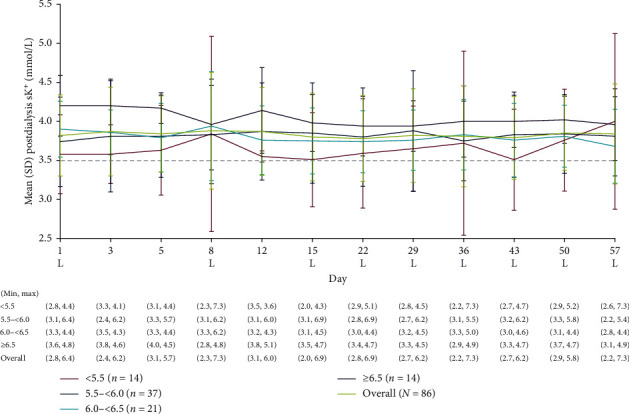
Postdialysis sK^+^ concentration at each study visit in placebo patients overall and by predialysis sK^+^ concentration at randomization. Predialysis sK^+^ measurement at randomization (visit 4, day 1) was used for subgroup stratification. No imputation of missing data was conducted. The dashed line represents the threshold for hypokalemia (i.e., sK^+^ 3.5 mmol/L). L represents the LIDI visits. LIDI, long interdialytic interval; SD, standard deviation; sK^+^, serum potassium.

At randomization, mean (SD) postdialysis sK^+^ concentration was 3.6 (0.5) mmol/L, 3.7 (0.6) mmol/L, 3.9 (0.4) mmol/L, and 4.2 (0.4) mmol/L in the randomization predialysis sK^+^ subgroups of <5.5 mmol/L, 5.5 to <6.0 mmol/L, 6.0 to <6.5 mmol/L, and ≥6.5 mmol/L, respectively. Mean (SD) postdialysis sK^+^ concentrations at day 57 were similar to randomization values: 4.0 (1.1) mmol/L, 3.8 (0.5) mmol/L, 3.7 (0.5) mmol/L, and 4.0 (0.5) mmol/L, respectively (Figure 4). Minor fluctuations from randomization at the end of the study in mean postdialysis sK^+^ concentration corresponded with fluctuations in predialysis sK^+^ concentration.

### 3.4. Shift in sK^+^ From Pre- to Postdialysis

**Figure 5:**
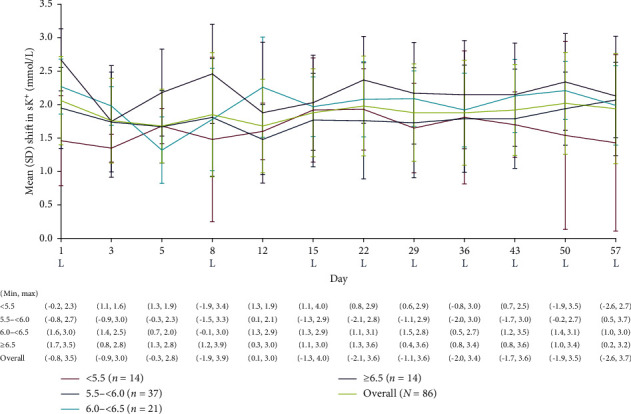
Shift in sK^+^ concentration from predialysis to postdialysis at each study visit in placebo patients overall and by sK^+^ concentration at randomization. Predialysis sK^+^ measurement at randomization (visit 4, day 1) was used for subgroup stratification. sK^+^ shift = predialysis sK^+^ − postdialysis sK^+^ for each visit. No imputation of missing data was conducted. L represents the LIDI visits. LIDI, long interdialytic interval; SD, standard deviation; sK^+^, serum potassium.

At randomization, mean (SD) shift in sK^+^ concentration was 1.5 (0.7) mmol/L, 2.0 (0.6) mmol/L, 2.3 (0.4) mmol/L, and 2.7 (0.5) mmol/L in the randomization predialysis sK^+^ subgroups of <5.5 mmol/L, 5.5 to <6.0 mmol/L, 6.0 to <6.5 mmol/L, and ≥6.5 mmol/L, respectively (Figure 5). Mean (SD) shift in sK^+^ at day 57 was similar to randomization values, with the biggest change from randomization in the most hyperkalemic patients: 1.4 (1.3) mmol/L, 2.1 (0.6) mmol/L, 2.0 (0.6) mmol/L, and 2.1 (0.9) mmol/L, respectively (Figure 5).

### 3.5. Probability of Recurrent Hyperkalemia

The risk of placebo patients having predialysis sK^+^ concentrations >5.5 or >6.0 mmol/L at LIDI visits during the 4-week evaluation period, determined as the proportion of hyperkalemic events divided by the total number of possible events, was 64.3% (*n* = 202/314) and 27.7% (*n* = 87/314), respectively. This risk increased among patients who were most hyperkalemic at randomization (Table 2). Patients with the lowest (<5.5 mmol/L) and highest (≥6.5 mmol/L) predialysis sK^+^ concentrations at randomization had a 43% and 80% chance, respectively, of having predialysis sK^+^ >5.5 mmol/L, and a 16% and 61% chance, respectively, of having predialysis sK^+^ >6.0 mmol/L at a LIDI visit during the 4-week evaluation period (Table 2).

### 3.6. Dialysis Parameters

**Figure 6:**
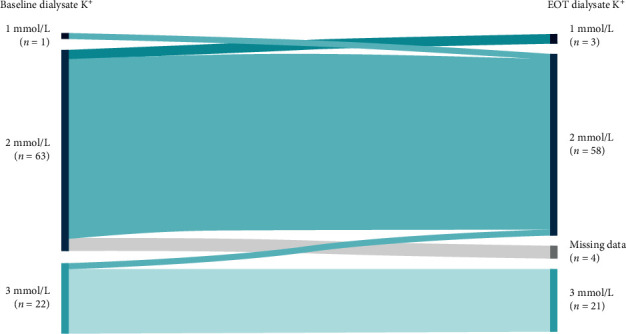
Shift in dK^+^ concentration category of 1, 2, and 3 mmol/L from baseline (visit 1, day −7) to the end of treatment (visit 15, day 57) in placebo patients. No imputation of missing data was conducted. Only subjects with visit 4 predialysis sK^+^ measurement were included. dK^+^, dialysate potassium; EOT, end of treatment; sK^+^, serum potassium.

Dialysis adequacy and prescription parameters at day 57 were generally comparable with baseline levels among placebo patients (*n* = 86) (Table 3). In particular, the mean (SD) dK^+^ concentration at day −7 (2.24 [0.46] mmol/L) was similar to day 57 (2.24 [0.46] mmol/L) (Table 3). When analyzed using dK^+^ concentration categories of 1, 2, and 3 mmol/L, from day −7 to day 57, dK^+^ concentration remained the same in the majority of placebo patients, increased in one patient, and decreased in four patients (Figure 6).

## 4. Discussion and Conclusions

These novel analyses utilized the placebo group of a randomized, double-blind phase 3b study to generate the first longitudinal trajectory of sK^+^ concentration among hyperkalemic HD patients. The mean predialysis sK^+^ concentration did not return to normokalemic levels (i.e., 4.0–5.0 mmol/L) in the placebo group overall and across all baseline predialysis sK^+^ subgroups over 8 weeks, with no change in dK^+^ concentration.

Evidence of longitudinal trajectory of sK^+^ concentration and risk of recurrent hyperkalemia following a hyperkalemic event is sparse [9–14], particularly among HD patients [1]. Among maintenance HD patients, a prospective French 2-year survey by Rossignol et al. reported that 26.4%, 13.8%, and 4.9% of patients had predialysis sK^+^ concentrations of >5.1 mmol/L, >5.5 mmol/L, and >6.0 mmol/L, respectively, and 73.8%, 57.9%, and 34.5% of patients had predialysis sK^+^ concentrations of >5.1 mmol/L, >5.5 mmol/L, and >6.0 mmol/L, respectively, during follow-up [1]. Furthermore, only 6.3% of patients became normokalemic within 3 months following a hyperkalemic event (sK^+^ >5.5 mmol/L) [1]. Adelborg et al. followed sK^+^ trajectories before and after a first hyperkalemic event among a broad population, which included patients with chronic kidney disease, although findings in dialysis patients alone were not presented separately [13]. In patients with a single hyperkalemic event, sK^+^ returned to prehyperkalemia concentrations in 2–4 weeks, while the reduction in sK^+^ concentrations in patients with repeated hyperkalemia occurred more slowly and did not reach the prehyperkalemia concentration [13]. Our findings in maintenance HD patients build on previous data to provide information on the likely trajectory of predialysis sK^+^ concentration following a hyperkalemic event and support the persistence of hyperkalemia reported previously. Importantly, as an sK^+^ concentration of >5.5 mmol/L is associated with increased mortality and hospitalization in HD patients [3–6], the patients in our analysis would be left at continued risk of adverse events.

In our analyses, more severe baseline hyperkalemia was associated with lower proportions of patients achieving sK^+^ ranges of 4.0–5.0 mmol/L and 4.0–5.5 mmol/L over 8 weeks. Furthermore, the risk of a subsequent hyperkalemic event (predialysis sK^+^ concentration >5.5 or >6.0 mmol/L) at LIDI visits during the 4-week evaluation period increased, with increasing baseline hyperkalemia. Similarly, Rossignol et al. determined the evolution of sK^+^ concentration among HD patients after an initial hyperkalemic event (sK^+^ >5.5 mmol/L) and reported that 80.2% and 59.7% of patients experienced a recurrent event of sK^+^ >5.1 mmol/L or of the same magnitude, respectively, within 3 months [1]. Furthermore, 35.6% of HD patients with an initial event of sK^+^ >6.0 mmol/L had a recurrent event of the same magnitude within 3 months [1]. Our findings among HD patients may inform physicians of the likely course of hyperkalemia based on a patient's baseline predialysis sK^+^ concentration. This is particularly important given the relationship between the greater severity of hyperkalemia and increased rates of mortality and hospitalization [9, 11, 14, 17].

Inadequate dialysis is associated with an increased predialysis sK^+^ concentration [18]. A recent position paper endorsed by the Italian Society of Nephrology stated that it is essential to evaluate dialysis adequacy in hyperkalemic patients [7]. Practice guidelines by the Kidney Disease Outcomes Quality Initiative (KDOQI) recommend a spKt/V of 1.4 per HD session for patients treated TIW, with a minimum delivered spKt/V of 1.2 [19]. In our analysis, the spKt/V exceeded the target level of 1.4 for patients receiving HD TIW as recommended by KDOQI [19], and parameters of dialysis adequacy and prescription were generally stable over time. Therefore, the impact of this variable on longitudinal changes in sK^+^ was minimized.

The significance of postdialysis sK^+^ concentration is less well-defined than for predialysis sK^+^ concentration, partly because postdialysis values are not routinely measured in clinical practice [15]. Our observed trends in longitudinal postdialysis sK^+^ concentration were not as clear as with predialysis sK^+^ concentration. Mean postdialysis sK^+^ concentration over 8 weeks remained above the threshold for hypokalemia (i.e., 3.5 mmol/L) in all baseline predialysis sK^+^ subgroups. However, normal postdialysis sK^+^ concentration may not prevent certain patients with marked hyperkalemia from a rebound of sK^+^ to potentially dangerous levels. A previous study noted a close correlation between predialysis and an increase in 6-hours postdialysis sK^+^ concentration (*r* = 0.78, *P* < 0.01) [8]. Furthermore, Rossignol et al. reported that ∼5% of HD patients who had an initial predialysis sK^+^ concentration >5.5 mmol/L were then hypokalemic (<3.5 mmol/L) within 3 months [1]. Together, these findings suggest a degree of potassium variability that deserves further attention.

The persistence of hyperkalemia observed in our analyses, despite maintenance HD, indicates that additional therapies and closer monitoring of HD patients following a hyperkalemic event may have a role in minimizing the risks of future hyperkalemia. The persistence of hyperkalemia reported by Rossignol et al. [1], despite widespread use of sodium polystyrene sulfonate, suggests that additional potassium binders are warranted. In the phase 3b DIALIZE study, SZC was shown to be an effective and well-tolerated treatment for predialysis hyperkalemia in patients with ESRD undergoing maintenance HD [16]. Data from real-world evaluations suggest that the potassium binder patiromer may also be effective at reducing sK^+^ concentration in HD patients [20, 21].

Our analyses have several limitations. The analyses are post hoc in nature and were not prespecified; therefore, the results are exploratory and hypothesis-generating. The patient numbers within the predialysis sK^+^ subgroups were small, which limits the interpretation of these findings. The impact of rescue therapy on mean sK^+^ concentration in placebo patients was not determined; however, in DIALIZE, a low proportion of placebo patients (5.1% [*n* = 5/99]) needed rescue therapy to reduce the sK^+^ concentration [16]. For the calculation of the risk of recurrent hyperkalemia, the definition of hyperkalemia differed from the stricter version used for study entry. Furthermore, the impact of a placebo effect resulting from clinical trial participation is unknown. Finally, rebound hyperkalemia is likely to be more severe in patients with shorter and less adequate HD, although this could not be assessed as patients received adequate HD.

In conclusion, mean predialysis sK^+^ concentration remains high over 8 weeks following a hyperkalemic event in patients receiving maintenance HD and routine dietary counselling. Most placebo patients remained hyperkalemic during this period, and so they may be at continued risk of adverse events. These findings may inform physicians of the likely course of hyperkalemia. Nephrologists may need to monitor predialysis sK^+^ concentration in hyperkalemic HD patients more closely and consider therapies, such as potassium binders, to minimize the risks of future hyperkalemia.

## Figures and Tables

**Table 1:** Baseline (visit 1, day −7) characteristics of placebo patients.

Summary statistic	Overall, *N* = 86	Predialysis sK^+^ at randomization (visit 4, day 1) (mmol/L)			
		<5.5, *n* = 14	5.5 to <6.0, *n* = 37	6.0 to <6.5, *n* = 21	≥6.5, *n* = 14
*Demographics*					
Age, years, mean (SD)	60.3 (13.2)	53.4 (12.3)	64.1 (11.1)	58.2 (14.7)	60.1 (14.7)
Age, years, *n* (%)					
18–50	18 (20.9)	6 (42.9)	5 (13.5)	4 (19.0)	3 (21.4)
51–64	28 (32.6)	4 (28.6)	11 (29.7)	8 (38.1)	5 (35.7)
65–84	39 (45.3)	4 (28.6)	21 (56.8)	8 (38.1)	6 (42.9)
≥85	1 (1.2)	0 (0)	0 (0)	1 (4.8)	0 (0)
Female sex, *n* (%)	36 (41.9)	3 (21.4)	15 (40.5)	11 (52.4)	7 (50.0)
Race, *n* (%)					
White	43 (50.0)	5 (35.7)	18 (48.6)	11 (52.4)	9 (64.3)
Black or African American	6 (7.0)	3 (21.4)	2 (5.4)	1 (4.8)	0 (0)
Asian	31 (36.0)	3 (21.4)	17 (45.9)	8 (38.1)	3 (21.4)
American Indian or Alaska Native	2 (2.3)	2 (14.3)	0 (0)	0 (0)	0 (0)
Other	4 (4.7)	1 (7.1)	0 (0)	1 (4.8)	2 (14.3)

*Clinical characteristics*					
Height, cm, mean (SD)	165.0 (9.2)	169.1 (10.1)	164.3 (7.9)	164.9 (11.8)	162.7 (5.9)
Weight, kg, mean (SD)	72.7 (16.4)	75.2 (15.0)	70.2 (15.3)	78.7 (20.1)	68.1 (12.0)
Body mass index, kg/m^2^, mean (SD)	26.7 (5.5)	26.2 (4.6)	25.9 (5.3)	28.9 (6.7)	25.7 (4.4)
Predialysis sK^+^, mmol/L, mean (SD)	6.0 (0.4)	5.9 (0.3)	5.9 (0.4)	6.0 (0.3)	6.3 (0.7)
Serum bicarbonate, mmol/L, mean (SD)	20.1 (2.4)	19.3 (1.9)	20.0 (2.2)	20.9 (3.3)	19.8 (1.9)

*Dialysis parameters*					
Vintage, years, mean (SD)	7.9 (7.8)	5.2 (3.9)	9.7 (8.8)	7.3 (8.9)	6.9 (4.8)
Duration of dialysis session, minutes, mean (SD)	241.7 (31.9)	236.1 (41.5)	245.7 (34.4)	242.8 (17.8)	235.1 (31.9)
Dialysate potassium, mmol/L, mean (SD)	2.2 (0.5)	2.1 (0.5)	2.2 (0.4)	2.3 (0.5)	2.4 (0.5)
Dialysate potassium, mmol/L, *n* (%)					
1	1 (1.2)	1 (7.1)	0 (0.0)	0 (0.0)	0 (0.0)
2	63 (73.3)	11 (78.6)	30 (81.1)	14 (66.7)	8 (57.1)
3	22 (25.6)	2 (14.3)	7 (18.9)	7 (33.3)	6 (42.9)
spKt/V, mean (SD)	1.7 (0.4)	1.5 (0.2)	1.7 (0.4)	1.8 (0.4)	1.9 (0.3)
Dialysate flow, mL/min, mean (SD)	548.0 (131.1)	583.6 (220.8)	563.4 (113.1)	521.9 (98.9)	511.1 (92.8)
Prescribed ultrafiltration rate, mL, mean (SD)	2785.8 (1228.1)	2426.6 (1665.1)	2929.8 (1250.4)	2871.4 (998.1)	2635.7 (996.6)
Urea removal rate, %, mean (SD)	74.3 (5.8)	71.8 (6.4)	74.1 (5.2)	74.1 (6.1)	75.9 (6.7)

*Key medical history, n (%)*					
Hypertension	69 (80.2)	11 (78.6)	31 (83.8)	16 (76.2)	11 (78.6)
Dyslipidemia	14 (16.3)	2 (14.3)	5 (13.5)	5 (23.8)	2 (14.3)
Cardiac failure	4 (4.7)	0 (0.0)	3 (8.1)	0 (0.0)	1 (7.1)
Diabetes mellitus	10 (11.6)	1 (7.1)	5 (13.5)	2 (9.5)	2 (14.3)
Type 1 diabetes mellitus	2 (2.3)	0 (0.0)	1 (2.7)	0 (0.0)	1 (7.1)
Type 2 diabetes mellitus	22 (25.6)	4 (28.6)	11 (29.7)	4 (19.0)	3 (21.4)

*Key allowed concomitant medications, n (%)*					
ACE inhibitors	12 (14.0)	3 (21.4)	4 (10.8)	3 (14.3)	2 (14.3)
Angiotensin II antagonists	23 (26.7)	2 (14.3)	16 (43.2)	3 (14.3)	2 (14.3)
Angiotensin II antagonists + calcium channel blockers	2 (2.3)	0 (0.0)	2 (5.4)	0 (0.0)	0 (0.0)
Aldosterone antagonists	3 (3.5)	0 (0.0)	2 (5.4)	1 (4.8)	0 (0.0)

ACE, angiotensin-converting enzyme; SD, standard deviation; spKt/V, target single pool Kt/V.

**Table 2:** Risk of predialysis sK^+^ concentration >5.5 or >6.0 mmol/L at LIDI visits during the 4-week evaluation period following the initial hyperkalemic event.

LIDI visit	Predialysis sK^+^ at randomization (visit 4, day 1) (mmol/L)			
	<5.5, *n* = 14	5.5 to <6.0, *n* = 37	6.0 to <6.5, *n* = 21	≥6.5, *n* = 14
*Incidence of predialysis sK * ^*+*^ * >5.5 mmol/L*				
Visit 12 (day 36)	8/13 (61.5)	19/33 (57.6)	10/18 (55.6)	11/13 (84.6)
Visit 13 (day 43)	4/12 (33.3)	18/33 (54.5)	15/20 (75.0)	10/13 (76.9)
Visit 14 (day 50)	5/12 (41.7)	23/33 (69.7)	17/21 (81.0)	12/13 (92.3)
Visit 15 (day 57)	5/14 (35.7)	24/33 (72.7)	13/21 (61.9)	8/12 (66.7)
**Overall**	**22/51 (43.1)**	**84/132 (63.6)**	**55/80 (68.8)**	**41/51 (80.4)**

*Incidence of predialysis sK * ^*+*^ * >6.0 mmol/L*				
Visit 12 (day 36)	2/13 (15.4)	3/33 (9.1)	4/18 (22.2)	8/13 (61.5)
Visit 13 (day 43)	1/12 (8.3)	4/33 (12.1)	6/20 (30.0)	6/13 (46.2)
Visit 14 (day 50)	3/12 (25.0)	10/33 (30.3)	9/21 (42.9)	10/13 (76.9)
Visit 15 (day 57)	2/14 (14.3)	10/33 (30.3)	2/21 (9.5)	7/12 (58.3)
**Overall**	**8/51 (15.7)**	**27/132 (20.5)**	**21/80 (26.3)**	**31/51 (60.8)**

Data are shown as the number (%) of hyperkalemic events. Hyperkalemia was defined as sK^+^ >5.5 or >6.0 mmol/L after the LIDI. No imputation of missing data was conducted. For each randomization predialysis sK^+^ subgroup, the probability of whether patients with hyperkalemia will be hyperkalemic (sK^+^ >5.5 or >6.0 mmol/L) at subsequent visits was calculated as follows: [Number of hyperkalemic events/total number of possible hyperkalemic events based on patients with evaluable data] × 100. LIDI, long interdialytic interval; sK^+^, serum potassium.

**Table 3:** Dialysis adequacy and prescription parameters in placebo patients (*n* = 86).

Day	spKt/V	Urea removal rate (%)	Dialysate flow (mL/min)	Dialysate potassium concentration (mmol/L)	Prescribed ultrafiltration rate (mL)	Blood flow (mL/min)
−7	1.71 (0.38)	74.3 (5.8)	548.0 (131.1)	2.24 (0.46)	2785.8 (1228.1)	321.0 (95.8)
1	1.99 (0.85)	73.3 (6.4)	545.3 (133.7)	2.23 (0.48)	2753.0 (1197.1)	316.0 (95.8)
3	—	—	—	2.24 (0.48)	—	—
5	—	—	—	2.23 (0.48)	—	—
8	—	—	—	2.22 (0.47)	—	—
12	—	—	—	2.22 (0.47)	—	—
15	—	—	—	2.24 (0.48)	—	—
22	—	—	—	2.26 (0.57)	—	—
29	1.67 (0.35)	72.3 (7.1)	545.9 (143.3)	2.21 (0.47)	2807.6 (1176.5)	317.3 (95.0)
36	—	—	—	2.22 (0.47)	—	—
43	—	—	—	2.22 (0.48)	—	—
50	—	—	—	2.22 (0.48)	—	—
57	1.70 (0.43)	73.6 (5.4)	548.7 (130.4)	2.24 (0.46)	2843.0 (1098.3)	320.7 (98.8)

Data are mean (standard deviation). Data are shown for the 86 placebo patients with predialysis sK^+^ measurements at randomization (visit 4, day 1). sK^+^, serum potassium; spKt/V, target single pool Kt/V.

## Data Availability

Data underlying the findings described in this manuscript may be obtained in accordance with AstraZeneca's data sharing policy described at https://astrazenecagroup-dt.pharmacm.com/DT/Home.
